# Analysis of a cohort of 279 patients with hairy-cell leukemia (HCL): 10 years of follow-up

**DOI:** 10.1038/s41408-020-0328-z

**Published:** 2020-05-27

**Authors:** Jerome Paillassa, Edouard Cornet, Stephanie Noel, Cecile Tomowiak, Stephane Lepretre, Sandrine Vaudaux, Jehan Dupuis, Alain Devidas, Bertrand Joly, Charlotte Petitdidier-Lionnet, Stephanie Haiat, Clara Mariette, Catherine Thieblemont, Didier Decaudin, Patricia Validire-Charpy, Bernard Drenou, Jean-Claude Eisenmann, Mario Ojeda Uribe, Agnès Olivrie, Mohamed Touati, Olivier Lambotte, Olivier Hermine, Jean-Michel Karsenti, Pierre Feugier, Willy Vaillant, Jean Gutnecht, Eric Lippert, Fabienne Huysman, Kamel Ghomari, Marouane Boubaya, Vincent Levy, Jeremie Riou, Gandhi Damaj, Aline Tanguy-Schmidt, Mathilde Hunault-Berger, Xavier Troussard

**Affiliations:** 1 Service des Maladies du Sang, CHU d’Angers, Angers, France; 2 Laboratoire d’Hematologie Biologique, CHU de Caen, Caen, France; 3 Service d’Oncologie Hematologique et Therapie Cellulaire, CHU de Poitiers, et CIC Inserm U1402, Poitiers, France; 4 Inserm U1245 et Service d’Hematologie, Centre Henri Becquerel et Normandie Univ UNIROUEN, Rouen, France; 5 Service d’Hematologie Clinique, CHU Henri Mondor, Assistance Publique des Hopitaux de Paris, Creteil, France; 6 Service d’Hematologie Clinique, CH Sud Francilien, Corbeil Essonnes, France; 7 Service d’Hematologie, CHU de Grenoble, Grenoble, France; 8 Service Hemato-Oncologie, Hopital Saint Louis, Assistance Publique des Hopitaux de Paris, Paris, France; 9 Unite d’Investigation Clinique, Departement de Medecine Oncologique, Institut Curie, Paris, France; 10 Service d’Hematologie Clinique, Institut Curie, Paris, France; 11 Service d’Hematologie Clinique, Groupe Hospitalier Regional de Mulhouse, Mulhouse, France; 12 Service d’Hematologie Clinique et Therapie Cellulaire, CHU de Limoges, Limoges, France; 13 Service de Medecine Interne et Immunologie Clinique, Hopital Bicêtre, Assistance Publique des Hopitaux de Paris, Paris, France; 14 Service d’Hematologie Adulte, Hopital Necker-Enfants Malades, Assistance Publique des Hopitaux de Paris, Paris, France; 15 Service d’Hematologie Clinique, CHU de Nice, Nice, France; 16 Service d’Hematologie, CHRU de Nancy, Nancy, France; 17 Service de Medecine Interne, Maladies Infectieuses, Oncologie et Hematologie, CH d’Auch, Auch, France; 18 Service de Medecine Interne, CHI Frejus Saint Raphaël, Frejus, France; 19 Laboratoire d’Hematologie Biologique, CHU de Brest, Brest, France; 20 Service d’Hematologie Oncologie, CH de Beauvais, Beauvais, France; 21 Unite de Recherche Clinique, Hopital Avicenne, Assistance Publique des Hopitaux de Paris, Bobigny, France; 22 MINT UMR INSERM 1066, CNRS 6021, Universite d’Angers, Angers, France; 23 Institut d’Hematologie de Basse-Normandie, CHU de Caen, Caen, France; 24 Federation Hospitalo-Universitaire ‘Grand Ouest Against Leukemia’ (FHU GOAL), Angers, France; 25 UFR Sante, Universite d’Angers, Angers, France; 26 CRCINA, INSERM, Universite de Nantes, Universite d’Angers, Angers, France

**Keywords:** Disease-free survival, Adverse effects, Hairy cell leukaemia, Chemotherapy, Risk factors

## Abstract

In total, 279 patients with hairy-cell leukemia (HCL) were analyzed, with a median follow-up of 10 years. Data were collected up to June 2018. We analyzed responses to treatment, relapses, survival, and the occurrence of second malignancies during follow-up. The median age was 59 years. In total, 208 patients (75%) were treated with purine analogs (PNAs), either cladribine (159) or pentosatin (49), as the first-line therapy. After a median follow-up of 127 months, the median overall survival was 27 years, and the median relapse-free survival (RFS) was 11 years. The cumulative 10-year relapse incidence was 39%. In patients receiving second-line therapy, the median RFS was 7 years. For the second-line therapy, using the same or another PNA was equivalent. We identified 68 second malignancies in 59 patients: 49 solid cancers and 19 hematological malignancies. The 10-year cumulative incidences of cancers, solid tumors, and hematological malignancies were 15%, 11%, and 5.0%, respectively, and the standardized incidence ratios were 2.22, 1.81, and 6.67, respectively. In multivariate analysis, PNA was not a risk factor for second malignancies. HCL patients have a good long-term prognosis. PNAs are the first-line treatment. HCL patients require long-term follow-up because of their relatively increased risk of second malignancies.

## Introduction

Hairy cell leukemia (HCL) is a rare B-cell chronic lymphoproliferative disorder characterized by atypical lymphoid cells with hairy projections in the peripheral blood, bone marrow, spleen, and/or liver^1,2^. HCL is responsible for 2% of leukemias^3^. Splenectomy^4^ and interferon-alpha (IFNα)^5^ were the first-line treatments, and purine analogs (PNAs), either cladribine^6^ or pentostatin^7^, were subsequently introduced. HCL prognosis improved consistently over time, with a 10-year overall survival (OS) of 90% with PNA treatment^8^. However, the management of HCL patients remains under investigation, particularly the care of patients with relapsed/refractory disease and the evaluation of the risk of second malignancies. For the first relapse, using the same PNA or switching to another PNA may be effective^9^. Anti-CD20 monoclonal antibodies (rituximab), alone^10^ or associated with PNA^11^, can also be alternative treatments for relapses. With the recent identification of the *BRAF V600E* mutation in most classic HCL (HCLc)^12^, BRAF inhibitors, namely, vemurafenib or dabrafenib, could be indicated^13,14^. MEK inhibitors (trametinib)^15^, BCR pathway inhibitors (ibrutinib)^16^, and anti-CD22 immunotoxins (moxetumomab pasudotox)^17^ are the newest therapeutic alternatives. The risk of second malignancies occurring during follow-up in HCL patients is controversial, with some studies describing a higher risk of cancer than in the general population^18–20^ and others describing no increased risk^21–24^. The reasons for these discrepancies might be the variability in the methods used to define second malignancies with pooling of second cancers occurring before and after HCL diagnosis in some studies^25^.

To answer this question, we investigated a large cohort of 279 HCL patients, with a 10-year median follow-up period, and we analyzed the treatments, responses, survival, relapses, and occurrence of second cancers.

## Subjects and methods

### Patients

The eligibility criteria for the HCL diagnosis were established according to the WHO 2008 and 2016 classifications, including morphological and flow cytometric analyses of blood, bone marrow or tissue specimens. Each patient signed an informed consent form. The study was performed in accordance with the Declaration of Helsinki.

### Study design and data collection

In the first analysis, we collected data up to 2012^26^. We then updated the data up to June 2018. A questionnaire was sent to the physicians, who were members of the French National Society of Hematology (SFH), with requests for the following data: date of last observation; last disease status: complete response (CR), partial response (PR), or progressive disease (PD); date of relapse(s); treatments and responses (CR, PR, failure); treatment start and end dates; second solid cancers (date, histology); hematological malignancies (date, WHO 2016 classification); death (date, cause); and other complications. The second cancers were defined as either synchronous cancers or metachronous cancers. Synchronous cancers were cancers that occurred at the same time as the diagnosis of HCL and those occurring within two months, as recommended by the Surveillance, Epidemiology, and End Results (SEER) Program. Metachronous cancers were defined by cancers occurring more than two months after the diagnosis of HCL. Overall survival (OS) was defined as the time from the date of HCL diagnosis until death from any cause or the date of the last observation. Relapse-free survival (RFS) was defined as the time from the start of treatment until relapse or death, and patients who remained free from disease were censored at the date of the last observation. Excel^®^ and FileMaker^®^ software were used for data collection.

### Treatments and evaluation of outcomes

Single-agent therapies included cladribine, pentostatin, IFNα, and rituximab. Splenectomy was also considered a single treatment modality if not associated with adjuvant drug therapy. Multiple-agent therapies were defined by the use of more than one drug within a period of 6 months. Responses were defined according to the *Consensus Resolution*^27^. CR required the morphologic absence of hairy cells in peripheral blood and bone marrow aspiration or biopsy specimens and the normalization of any organomegaly and cytopenia. Immunophenotypic analysis of peripheral blood or bone marrow biopsy was not required. PR was defined as the normalization of the peripheral counts associated with at least a 50% reduction in organomegaly and bone marrow hairy cells and <5% circulating hairy cells. All other outcomes were considered non-responses. Relapse was defined as any deterioration in blood counts related to the detection of hairy cells in the peripheral blood and bone marrow.

### Statistical analyses

Survival curves were drawn according to the Kaplan–Meier method. OS and RFS were compared using the log-rank test. The cumulative incidence of relapse (CIR) and cumulative incidence of second cancer were evaluated considering death as a competing risk. The CIRs were compared according to the first-line treatment with Gray’s test. Multivariate analyses of OS and RFS were performed using the Cox regression model, and multivariate analyses for the cumulative incidence of second malignancies were performed using the Fine and Gray^28^ regression model, considering death as a competing risk. An excess of second malignancies was expressed by the standardized incidence ratio (SIR), which was defined as the ratio between the number of observed and expected cases from the general population in France. The expected malignancy rates were calculated from the age-indexed (in 5-year categories) cancer incidence in France^29,30^. These incidence rates were multiplied by the observed person-years at risk in each age category to calculate the expected frequency of second malignancies. Confidence intervals (CIs) of the SIR were obtained by assuming a Poisson distribution for the observed numbers. The SIR was calculated for second cancers, second solid cancers, and second hematological malignancies. As the French registries did not include nonmelanoma skin cancers or monoclonal gammopathies of undetermined significance/monoclonal gammopathies of clinical significance (MGUS/MGCS), we did not take into account these malignancies in the “observed cases” to calculate the SIR. SPSS^®^ (version 16.0) and R^®^ (version 3.5.3) were used for the statistical analyses.

## Results

### Patient characteristics

Two hundred and seventy-nine patients from 19 French centers were analyzed, with a median follow-up of 127 months (range 2–413). All the patients had HCL diagnosed between 1980 and 2011. The characteristics of the 279 patients are listed in Table 1. The median age at HCL diagnosis was 59 years (range 29–88). Twenty-one percent of patients presented had an infectious disease at the time of the diagnosis of HCL. The median hemoglobin (Hb) level was 12 g/dL, the median platelet count was 93.5 × 10^9^/L, the median white blood cell count was 2.68 × 10^9^/L, and the median neutrophil count was 0.99 × 10^9^/L. The median percentage of bone marrow hairy cells evaluated in the bone marrow aspirations was 3% (range 0–94). Sixty-one percent of patients underwent flow cytometry analysis at diagnosis. There were personal histories of cancer in 31 patients, representing 11% of the patients (Supplementary Information 1). The median time between the personal history of cancer and the diagnosis of HCL was 4 years (range 0–44). There were 85 familial histories of malignancy (69 solid cancers and 16 hematological malignancies) in 63 patients, representing 23% of the patients (Supplementary Information 2). In total, 45, 15, and 3 patients had 1, 2, and 3 family members with histories of malignancy, respectively.

**Table 1:** Patient characteristics at baseline.

Age at HCL diagnosis (years), median [range]	59 [29–88]
Hemoglobin (g/dL), median [range]	12 [3–16.7]
Platelet count (×10^9^/L), median [range]	93.5 [7.4–503]
White blood cell count (×10^9^/L), median [range]	2.68 [0.5–107.2]
Neutrophil count (×10^9^/L), median [range]	0.99 [0.027–12.96]
Hairy cells (%), median [range]	3 [0–94]
Flow cytometry analysis, *n* (%)	170 (61)
CD25^+^, *n* (%)	118 (69)
CD103^+^, *n* (%)	123 (72)
CD11c^+^, *n* (%)	109 (64)
Infectious disease at diagnosis, *n* (%)	58 (21)

### New events

New events were defined as new relapses, death or second cancers occurring since the first analysis. In total, 99/279 patients (36%) experienced at least one new event. We observed 130 new events: 60 relapses (1 new relapse in 54 patients (19% of patients), 2 new relapses in 6 patients (2%)), 25 solid second cancers, 12 s hematological malignancies, and 33 new deaths. At the last follow-up, 229 patients were still alive: 193 were in CR (84%), 19 were in PR (8%), 10 had PD (5%), and 7 had an unknown disease status (3%) (Supplementary Information 3).

### PNAs are the treatment of choice for first-line treatment and treatment of the first relapse

The median number of lines of treatment was 1 (range 0–7). The treatments received in each line of therapy are shown in Table 2.

**Table 2:** Treatments according to the line of treatment.

1st line treatment	*n* = 279 (100%)
PNA	208 (75)
Cladribine	159 (57)
Pentostatin	49 (18)
Other	59 (21)
IFN	40
IFN then cladribine	7
Splenectomy	3
Cladribine + rituximab	2
Pentostatin then cladribine	2
Splenectomy then pentostatin	1
IFN then pentostatin	1
R-CHOP	1
Pentostatin + rituximab	1
Unknown	1
No treatment	12 (4)

2nd line treatment	*n* = 112 (100%)
PNA	77 (69)
Cladribine	59 (53)
Pentostatin	18 (16)
Other	25 (22)
IFN	11
Rituximab	7
Cladribine + rituximab	3
Pentostatin + rituximab	1
IFN + rituximab	1
IFN then pentostatin	1
Splenectomy	1
No treatment	10 (9)

3rd line treatment	*n* = 49 (100%)
PNA	29 (59)
Cladribine	12 (24)
Pentostatin	17 (35)
Other	15 (31)
Rituximab	5
Cladribine + rituximab	4
IFN	3
Pentostatin + rituximab	1
Cladribine + IFN	1
Rituximab + bendamustine then vemurafenib	1
No treatment	5 (10)

4th line treatment	*n* = 16 (100%)
PNA	9 (56)
Cladribine	6 (38)
Pentostatin	3 (18)
Other	7 (44)
IFN	3
Rituximab	2
Pentostatin + rituximab	1
IFN + rituximab	1

5th line treatment	*n* = 9
PNA	5
Cladribine	3
Pentostatin	2
Other	4
IFN	2
Fludarabine + rituximab	1
R-CHOP	1

6th line treatment	*n* = 5
PNA	2
Cladribine	1
Pentostatin	1
Other	2
IFN	1
Splenectomy	1
No treatment	1

7th line treatment	*n* = 4
PNA	1
Cladribine	1
Other	3
IFN	2
R-DHAX then HSCT	1

“then” means that patients received a sequential treatment.

*PNA* purine analogs, *IFN* interferon α, *R-CHOP* rituximab, cyclophosphamide, doxorubicin, vincristine, and prednisolone, *R-DHAX* rituximab, dexamethasone, cytarabine, and oxaliplatin, *HSCT* hematopoietic (allogeneic) stem cell transplantation.

As a first-line therapy, 208 patients (75%) received PNA, either cladribine (159 patients: 57%) or pentostatin (49 patients: 18%). Fifty-nine patients received various other treatments (21%), with 40 patients treated with IFNα (14%) (Table 2). Twelve patients (4%) never received any treatment. Only the year of HCL diagnosis significantly influenced the first-line treatment (more patients were treated with PNA after 2000 (*p* < 0.001)), which was not the case for age, infection, Hb level, platelet count, or neutrophil count at diagnosis.

With regard to second-line therapies, 77 patients (69%) were re-treated with PNAs: 59 patients with cladribine (53%) and 18 with pentostatin (16%). Twenty-five patients (22%) received other treatments, with 11 patients receiving IFNα (10%). Ten patients (9%) did not receive treatment for a relapse.

The subsequent lines of treatment were heterogeneous (Table 2). Forty-four patients (16%) received 3 lines of treatment, 16 patients (6%) received 4 lines, 9 patients (3%) received 5 lines, 4 patients (1%) received 6 lines, and 4 patients (1%) received 7 lines of treatment. Only one patient received 4 cycles of vemurafenib (960 mg bid 21 days/28) as a third-line therapy, which was stopped after achieving a CR and developing invasive pulmonary aspergillosis. Two years after stopping vemurafenib, the patient was still in CR.

### PNA induced the highest CR rate and duration of response (DOR)

The overall response rates (ORRs), including CR and PR after first-line treatment, were 99% for all patients, 100% for patients treated with PNA, 99% for those treated with cladribine, 100% for those treated with pentostatin, 96% for those treated with other treatments (including IFNα), and 90% for those treated exclusively with IFNα. The CR rates for the patients were 78%, 83%, 83%, 84%, 58%, and 50%, respectively. The CR rate was significantly lower for patients who received other treatments or IFNα (*p* < 0.001) (Supplementary Information 4).

The DOR (median (min; max)) was 91 months (3; 335) for all patients who received first-line therapy, 94 months (4; 260) for those who received PNA, 94 months (8; 260) for those who received cladribine, 88 months (4; 251) for those who received pentostatin, and only 52 months (3; 335) for those who received other treatments. The difference in the median DOR among patients receiving cladribine, pentostatin and other treatments was statistically significant (*p* = 0.02). The median DOR also significantly decreased with the line of treatment (*p* < 0.001) (Supplementary Information 5), which remained significant when we only considered treatment with PNAs (*p* < 0.001).

### PNA provided the best RFS and the lowest CIR

Fifty patients died (Supplementary Information 1). Among the 33 known causes of death, second malignancies accounted for the most deaths (33%). The median OS was 27 years (328 months; 95% CI: 299; 357).

**Fig. 1:**
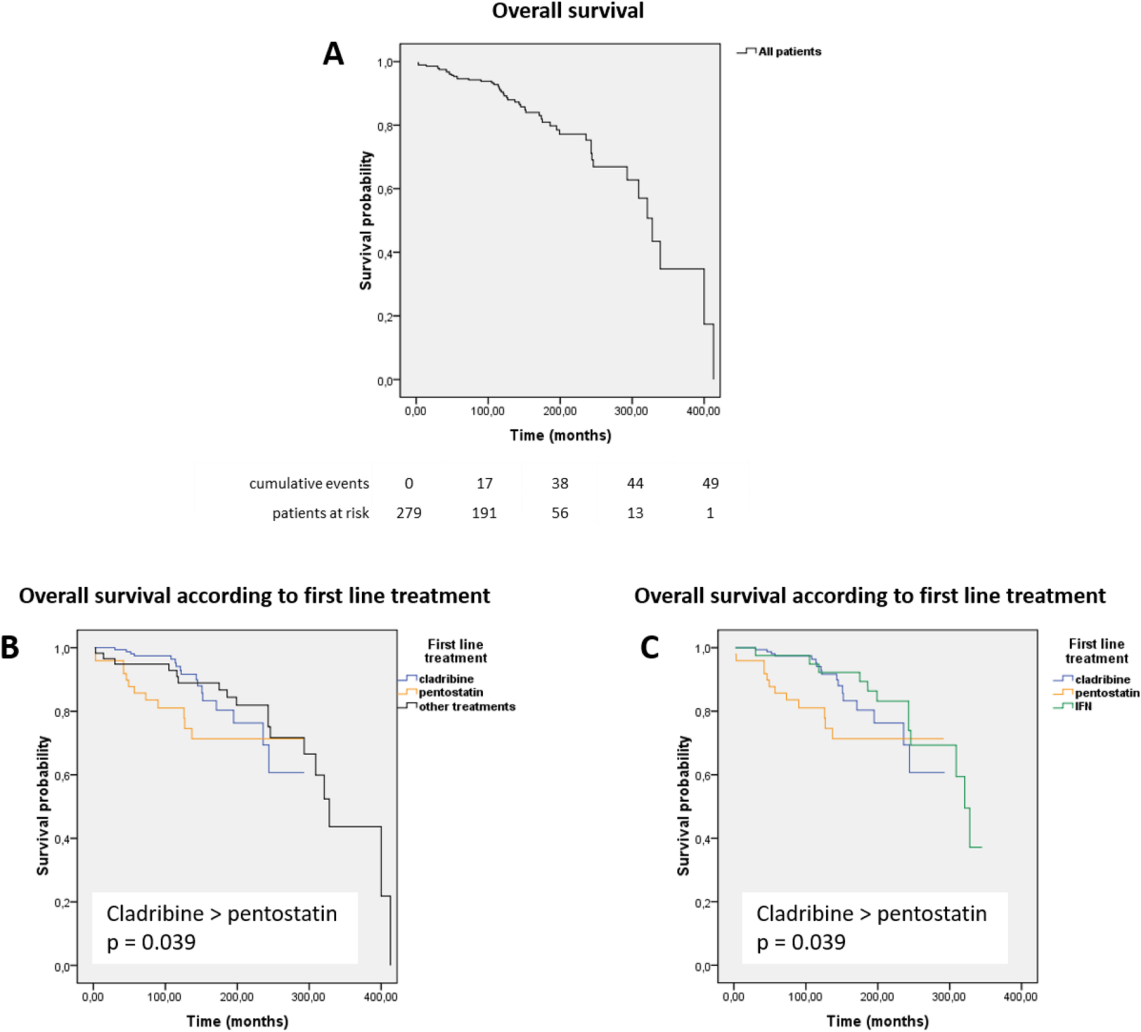
Overall survival. **a** all 279 HCL patients, **b** in line with the first-line treatment: cladribine, pentostatin, and other treatments including IFNα, **c** in line with the first-line treatment: cladribine, pentostatin, and IFNα. Kaplan–Meier method.

After excluding the 12 patients who never received any treatment and the patient for whom the first-line treatment was unknown, the median OS was not reached for patients who received cladribine or pentostatin, whereas it was 328 months (95% CI: 300–356) for patients treated with other treatments and 321 months (95% CI: 298–344) for patients treated with IFNα (Fig. 1). Patients receiving cladribine had a significantly better OS than patients receiving pentostatin (log rank test, *p* = 0.039). Indeed, the 5-year OS was 97% in patients receiving cladribine versus 86% in patients receiving pentostatin (Table 3). In multivariate analysis (Supplementary Information 6), only age at diagnosis was a predictor of an inferior OS (*p* < 0.001, HR = 1.082, 95% CI: 1.044–1.121).

**Table 3:** Results after first-line treatment.

	Cladribine	Pentostatin	“Other” treatments	All treated patients
Follow-up (months), median (range)	116 (21–293)	126 (2–292)	221 (3–413)	128 (2–413)
ORR, *n* (%)	157 (99)	45 (100)	52 (96)	255 (99)
CR, *n* (%)	131 (83)	38 (84)	32 (58)	201 (78)
Median RFS (months)	163	159	55	136
CIR				
1 year (%)	2.5	4.4	5.5	3.5
5 years (%)	14	13	49	21
10 years (%)	33	30	66	39
Median OS (months)	NR	NR	328	321
OS, evaluable patients				
1 year, *n* (%)	100 (158)	96 (47)	98 (57)	99 (263)
5 years, *n* (%)	97 (150)	86 (42)	95 (54)	95 (246)
10 years, *n* (%)	94 (77)	81 (25)	89 (43)	90 (145)
20 years, *n* (%)	69 (8)	71 (3)	82 (26)	76 (37)

The median RFS after first-line treatment was 11 years (136 months (95% CI: 109; 163)): 163, 159, 55, and 50 months after treatment with cladribine, pentostatin, other treatments and IFNα, respectively (Supplementary Information 7). Patients treated with cladribine or pentostatin as a first-line therapy had a significantly better RFS than patients treated with other treatments or IFNα (log rank test, *p* < 0.001). However, the difference in RFS between patients treated with cladribine and those treated with pentostatin was not statistically significant. In multivariate analysis (Supplementary Information 8), the percentage of hairy cells at diagnosis as a continuous variable (*p* = 0.006, HR 1.010, 95% CI: 1.003; 1.018) and using other first-line treatments (*p* = 0.004, HR 2.533, 95% CI: 1.349; 4.754) were both predictors of a worse RFS, whereas achieving CR1 (*p* < 0.001, HR 0.355, 95% CI: 0.228; 0.555) was a predictor of a longer RFS.

**Fig. 2:**
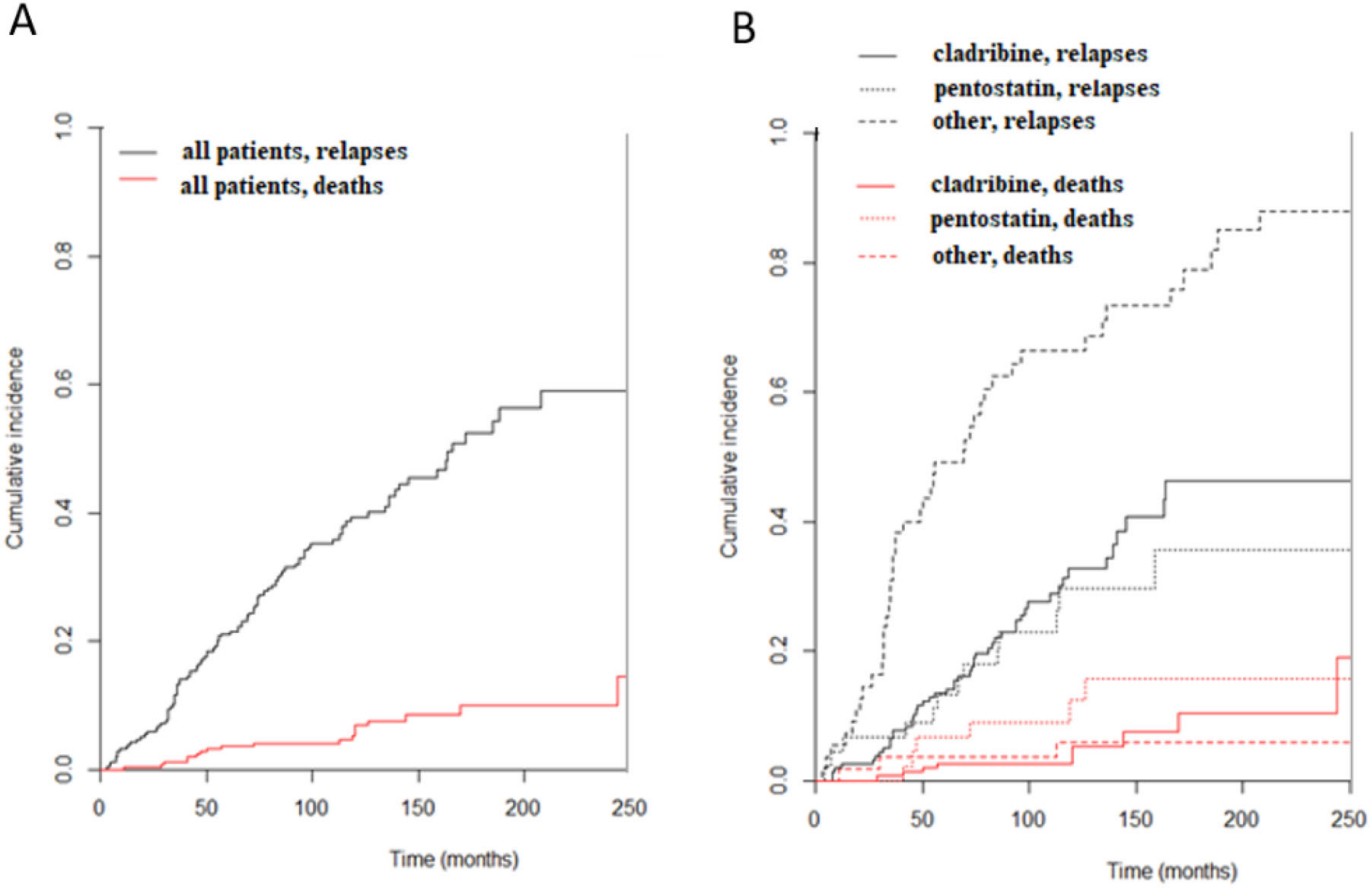
Cumulative incidence of relapse (CIR): **a** all patients who responded to first-line treatment, **b** according to first-line treatment.

The proportions of patients with relapses after the first, second, third, fourth, fifth, and sixth lines of treatment were 106/257 (41%), 49/102 (48%), 16/44 (36%), 9/16 (56%), 5/9 (56%), and 4/4 (100%), respectively. With regard to the first-line treatment, the proportions of patients with relapses after treatment with PNA, cladribine, pentostatin, and other treatments were 31%, 31%, 29%, and 80% (90% for IFNα), respectively. For all patients who responded to the first-line treatment, considering death as a competing risk, the CIR increased with time and was 3.5% (95% CI: 1.7; 6.3), 5.8% (95% CI: 3.4; 9.2), 21.1% (95% CI: 16.4; 26.4), and 39.4% (95% CI: 32.8; 45.9) at 1, 2, 5, and 10 years, respectively. Patients who received other treatments had a statistically significant higher CIR than patients who received cladribine or pentostatin (Gray’s test^31^, subdistribution hazard ratio (sdHR) = 44.6, *p* < 0.001) (Fig. 2).

The median RFS after second-line therapy (called RFS2) was 86 months (95% CI: 70; 102) for all patients. According to the second-line therapy used, the median RFS2 was 79, 116, and 40 months after cladribine, pentostatin, and other treatments, respectively. Patients who received pentostatin had a significantly longer RFS2 than patients who received other treatments (log-rank test, *p* = 0.050) (Supplementary Information 9). To test the effect of switching one PNA for another, we analyzed patients who received PNA in the first and second lines (*n* = 44, Supplementary Information 10). Among them, 31 patients were treated with the same PNA in both lines (no switching: 30 patients cladribine then cladribine; 1 patient pentostatin then pentostatin), whereas 13 patients switched PNAs (switching: 8 patients cladribine then pentostatin; 5 patients pentostatin then cladribine). Overall, 5/44 patients (11%) experienced a relapse (4 patients who had received cladribine then cladribine and 1 patient who had received pentostatin then cladribine). The median DOR was 48 months (range 4–120). There was no statistically significant difference in terms of the median DOR between patients who switched (52 months) and those who did not (36 months). The median RFS was 71 months. There was no statistically significant difference in terms of the median RFS between patients who switched (55 months) and those who did not (71 months) (Supplementary Information 11). Thus, for the second-line treatment, we found that pentostatin had an advantage. We did not observe a significant difference in outcomes between patients who switched and those who were re-treated with the same PNA according to these results based on the DOR and RFS.

Table 3 summarizes the results after first-line treatment.

### Risk of infectious or immune complications

Forty-three of 279 patients (15%) experienced at least one infection during follow-up. Lower and upper respiratory tract infections were the most frequent complications. Three patients experienced invasive pulmonary aspergillosis, including the patient treated with vemurafenib. A total of 7/279 patients (2.5%) had at least one immune complication. We observed the following complications: 3 with vasculitis, 1 with anti-MAG neuropathy with cryoglobulin, 1 with sarcoidosis, 1 with acute polyarthritis, 1 with rheumatoid arthritis, 1 with immune thrombocytopenia, and 1 with glomerulopathy with IgA mesangial deposits.

### Risk of second cancers

**Fig. 3:**
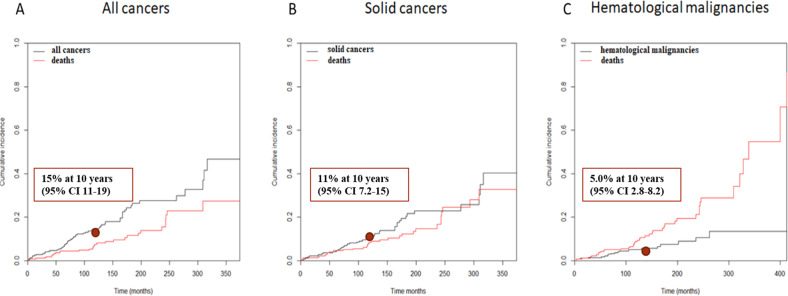
Cumulative incidence of **a** second cancer, **b** second solid cancers, **c** second hematological malignancies.

Twenty-one percent of patients (59/279) experienced at least 1 second cancer (68 s cancers), 17% (46/279) experienced at least 1 solid cancer (three of them had two successive solid cancers), and 6.8% (19/279) experienced a hematological malignancy. The most prevalent solid tumors were prostate and nonmelanoma skin cancers. The most prevalent hematological malignancies were MGUS/MGCS (Table 4). The patient treated with vemurafenib did not experience any second malignancy. The median times between the diagnosis of HCL and all second cancers, solid cancers and hematological malignancies were 81 months (range 0–374), 99 months (range 0–374), and 78 months (range 2–262), respectively. The median ages at the diagnosis of all second cancers, solid cancers or hematological malignancies were 70, 69, and 77 years, respectively. When comparing the occurrence of all second cancers, solid cancers and hematological malignancies according to first-line treatment, the differences were not statistically significant. Considering death as a competing risk, the 10-year cumulative incidences of all second cancers, solid cancers, and hematological malignancies were 15% (95% CI: 11; 19), 11% (95% CI: 7.2; 15), and 5.0% (95% CI: 2.8; 8.2), respectively (Fig. 3). Then, we performed univariate and multivariate analyses (Supplementary Information 12) using the Fine and Gray^28^ regression model, considering death as a competing risk, and including age at HCL diagnosis, a familial history of cancer, a personal history of cancer, treatment with cladribine (regardless of the line of treatment), treatment with pentostatin, and treatment with IFNα as covariates. In multivariate analysis, IFNα was a protective factor against second cancers (*p* = 0.038, sdHR 0.529, 95% CI: 0.290; 0.966), a familial history of cancer was a risk factor for solid cancers (*p* = 0.017, sdHR 2.117, 95% CI: 1.146; 3.910), and a personal history of cancer was a risk factor for hematological malignancies (*p* = 0.028, sdHR 3.473, 95% CI: 1.144; 10.550). Only 1/5 of the patients with a personal history of hematological malignancies developed a second hematological cancer during follow-up. Compared to the French population, our patients had an excess of cancers (SIR: 2.22; 95% CI: 1.61–2.83), solid cancers (SIR: 1.81; 95% CI: 1.24–2.38), and hematological malignancies (SIR: 6.67; 95% CI: 3.04–10.30).

**Table 4:** Second solid cancers (left) and second hematological malignancies (right) observed during the follow-up period.

Solid cancers	*n* = 49	Hematological malignancies	*n* = 19
Prostate	11	MGUS/MGCS	6
Nonmelanoma skin cancer	11	MDS	4
Lung	7	NHL^a^	3
Colorectal	7	MDS/MPN	2
Kidney	4	MPN	1
Pancreas	2	AML	1
Esophagus	1	MM	1
Pleural	1	CLL	1
Unknown	1		
Kaposi	1		
Biliary tract	1		
Bladder	1		
Breast	1		

^a^1 DLBCL, 1 FL, 1 SMZL.

*AML* acute myeloid leukemia, *MM* multiple myeloma, *CLL* chronic lymphocytic leukemia, *NHL* non-Hodgkin’s lymphoma, *DLBCL* diffuse large B-cell lymphoma, *FL* follicular lymphoma, *SMZL* splenic marginal zone lymphoma.

## Discussion

We analyzed 279 HCL patients, with a median follow-up of 10 years. HCL patients treated with PNA have a good long-term prognosis. However, relapses and second malignancies are common.

In our cohort, PNAs remained the first choice for first-line treatment and treatment of the first relapse. PNAs gave the best CR rate, DOR, and RFS, and were associated with the lowest relapse rate (Supplementary Information 13)^8,18,20,22,24,32–42^. Madanat et al.^8^ analyzed the data of 61 patients with cladribine as the first-line treatment. The ORR and CR rates were 97% and 78%, respectively. Nineteen patients relapsed, 12 of whom received second-line treatment with cladribine, resulting in an ORR of 83%^8^. Hacioglu et al.^37^ described a cohort of 94 HCL patients, most of whom received first-line treatment with cladribine. For the patients treated with cladribine, the ORR, CR and relapse rates were 97%, 81%, and 17%, respectively. Cladribine was the first treatment choice in the second and third lines, with CR rates of 68% and 67%, respectively^37^.

In our cohort, the responses were shorter at each relapse. Zinzani et al.^39^ evaluated the long-term outcomes of 121 patients: the CR rates after the first, second, third, fourth and fifth lines of treatment were 77, 74, 71, 65, and 50%, and the median DORs were 2.7, 2.5, 2.2, 1.6 and 1.3 years, respectively.

Our study confirmed the good long-term prognosis of HCL. The median OS was 27 years, which is in line with duration reported in the literature (Supplementary Information 13)^8,18,20,22,24,32–42^. In a study by Else et al.^38^ including 233 patients with long-term follow-up, the 15-year OS was 78%. In another study including 44 patients treated with cladribine, the 12-year OS was 79%^34^. Strikingly, in our cohort, we found that patients had a better OS with cladribine than with pentostatin, which was not the case in our first analysis or in the literature. However, we did not find any difference in OS between first-line treatments in multivariate analysis. One explanation could be that patients who received cladribine were in better condition than the patients treated with pentostatin. We compared the characteristics at baseline of patients stratified by the first-line therapy they received, but we did not find any statistically significant differences in terms of age or infectious disease at diagnosis between the treatment groups. However, because of the retrospective nature of this study, with data obtained via responses to a questionnaire, we had no information about the performance status, fitness or comorbidities of the patients. Only age at HCL diagnosis was a predictor of OS in multivariate analysis.

The median RFS was 11 years, which was in line with the RFS observed in previous cohorts (Supplementary Information 13)^8,18,20,22,24,32–42^. In a review of retrospective studies with a long-term follow-up including patients treated with PNA, the median RFS was 13–16 years^43^. In our cohort, we found that first-line treatment with PNAs, CR1 achievement, and a low percentage of hairy cells in the bone marrow at diagnosis were predictors of a better RFS in multivariate analysis. In a retrospective study comparing three first-line therapies in 71 patients (31 cladribine, 19 IFNα, 16 splenectomy), the progression-free survival (PFS) time was significantly longer and the relapse rate was lower for patients treated with cladribine than for those undergoing the other two treatments^33^. In a Spanish retrospective cohort of 107 patients, the median treatment-free interval (TFI) was shorter for patients in PR than for those in CR^36^. We confirmed that relapses remain an issue, with a 10-year CIR of 39% after first-line treatment. This highlights the issue of late relapses occurring several years after treatment. Moreover, PNAs were again found to be the best choice, resulting in a lower CIR than other treatments.

In our cohort, IFNα remained an important option; it was used in 40/279 patients (14%) as the first-line therapy and in 11/112 patients (10%) as the second-line therapy. The outcomes after treatment with IFNα were inferior to those obtained after treatment with PNAs in terms of the CR, RFS and relapse rates. However, there was no statistically significant difference in ORR or OS. In our opinion, IFNα should be the preferred option for patients with an active infection at diagnosis and those for whom PNAs are not an option.

We did not find any difference in the outcomes of patients who switched or did not switch PNAs between the first and second lines of treatment. In their review, Else et al.^43^ described no difference in CR rates between patients who did and did not switch PNAs. In a long-term follow-up of 233 patients with HCL initially treated with pentostatin or cladribine, the CR rates, relapse rates, PFS, and RFS were not significantly different between patients who did and did not switch treatments after relapsing^38^.

During follow-up, 15% of the patients experienced at least one infection. This was probably underestimated because infections were underdeclared. In a retrospective study, Damaj et al.^44^ included 73 patients, most of whom were treated with IFNα as a first-line treatment. With a median follow-up of 13 years, 37% of the patients experienced at least one infection, and 15% experienced a severe infection^44^. In our study, one patient developed tuberculosis, and three patients experienced pulmonary invasive aspergillosis. According to the literature, HCL patients have a higher risk of mycobacterial infections, but invasive fungal diseases (IFDs) have rarely been described^33,45–47^.

Our study also showed that patients with HCL had a risk of developing second cancers. This risk was higher than in the general population: the SIRs for all cancers, solid tumors, and hematological malignancies were 2.22, 1.81, and 6.67, respectively. Several studies also found that HCL patients have a higher risk of developing second cancer than the general population (SIR 1.24–4.33, Table 5). These studies also found relatively increased cancer-related mortality rates among HCL patients^18–20,48,49^. In our cohort, we found that second cancers were the primary cause of death. In some studies, the risk of second cancer was especially high when the cancer was a hematological malignancy, and this was also observed in our study^48,49^. We found that a personal history of cancer was a risk factor for hematological malignancies. Our hypothesis is that these patients have received chemotherapy/radiotherapy, favoring the development of hematological malignancies.

**Table 5:** (Part 1). Other HCL cohorts with cancer incidence during follow-up. (Part 2). Other HCL cohorts with cancer incidence during follow-up.

Study	Type	*n*	Treatments	Follow-up	Cumulative incidence of cancers	SIR or observed/expected ratio (95% CI)
Hisada et al.	Retrospective	3104		6.5 years	32% at 25 years^a^	1.24 (1.11; 1.37)
Kampmeier et al.	Prospective	69	IFNα	91 months	19%^a^	4.33
Goodman et al.	Retrospective	209	Cladribine	≥7 years	23%^a^	2.03 (1.49; 2.71)
Saven et al.	Retrospective	358	Cladribine	58 months	8%^a^	1.88 (1.24; 2.74)
Au et al.	Retrospective	117	Cladribine pentostatin splenectomy IFNα	68 months	24%^a^	2.60 (1.82; 3.61)
Paltiel et al.	Retrospective	181	Cladribine	80 months	11%^b^	1.3 (0.68; 2.28) for all cancers 3.23 (1.39; 6.36) for urogenital cancers
Kurzrock et al.	Retrospective	350	Cladribine pentostatin IFNα	6 years	7.40%^b^	1.34 for all cancers 13.04 for myelomas 8.7 for lymphomas
Federico et al.	Retrospective	1022			14% at 15 years^b^	1.01 (0.74; 1.33) for all cancers 5.3 (1.9; 11.5) for NHL
Else et al.	Retrospective	233	Pentostatin cladribine	16 years	12% (excluding nonmelanoma skin cancers)^c^	No data
Flinn et al.	Prospective	241	Pentostatin +/−IFNα	9.3 years	16%^c^	1.26 (0.86; 1.77)
Pawson et al.	Retrospective	200	Cladribine pentostatin IFNα	65 months	4%^c^	1.29 (0.60; 2.65)
Maloisel et al.	Retrospective	238	Pentostatin	63.5 months	7.60%^c^	0.95 (0.5; 1.92)
Rosenberg et al.	Retrospective	88	Cladribine	21 years	9.10%^c^	1.60 (0.80; 2.89)
Watts et al.	Retrospective	267	Cladribine pentostatin		11% at 10 years (melanoma and non-melanoma skin cancers only)^c^	1.30 (0.78; 2.03) (for melanoma only)
Troussard et al.	Retrospective	107	IFNα	102 months	9.5% at 10 years^c^	1.24 (0.54; 2.45)
Getta et al.	Retrospective	331	Cladribine splenectomy IFNα	69 months	Age ≤ 40: 21% at 10 years^d^ Age > 40: 29% at 10 years^d^	No data
Damaj et al.	Retrospective	73	Cladribine pentostatin splenectomy IFNα	13 years	27+/−6% at 13 years^d^	No data

^a^Excess of second malignancies.

^b^Excess for some cancers only.

^c^No excess of second malignancies.

^d^No data about the excess of risk compared to the general population.

In other studies, HCL patients did not have a higher risk of developing a second cancer (Table 5)^21–24,40,43,50^. Some studies found an excess of risk only for certain cancers (Table 5)^51–53^.

In our study, IFNα was a protective factor against cancer in multivariate analysis. However, cladribine and pentostatin were not risk factors for cancers, even if the results of our multivariate analysis should be interpreted with caution because most patients received PNAs. Of note, ten patients with a second cancer did not receive PNAs. Therefore, it seems that the risk of second malignancies might be related to HCL itself rather than to the treatments, which is in line with the findings of other studies^49^.

Our study has several strengths. First, the sample size was large considering the rarity of HCL. In addition, the follow-up was long, with a median of 10 years. Moreover, this study showed the “real-life” experience of treatments for HCL, with the inclusion of non-selected patients. Finally, it was a collaborative and multicentric study, including patients from 19 centers. However, the study has several limitations. First, it was a retrospective study with data obtained via responses to a questionnaire. Therefore, there was a risk of information bias. In addition, there was also a declaration bias regarding infections and autoimmune diseases. Indeed, the incidence of autoimmune complications was ten times lower than that reported in the literature (2.5% versus 25%)^54–56^. Moreover, we had no information about the proportion of HCLv patients, who have a worse prognosis than HCL patients. Regarding treatments, the number of cycles of pentostatin has rarely been reported. It is unknown whether the number of cycles has an impact on outcomes, second malignancies and infections. Regarding the evaluation of response, there was a lack of standardization among the different centers in terms of the type and timing of the evaluation, which led to a measurement bias. In addition, there was a lack of statistical power in our multivariate analyses for the cumulative incidences of solid cancers and hematological malignancies due to the lack of events. Then, many cancers were nonmelanoma skin cancers and MGUS/MGRS. Some authors did not take into account these types of cancer^53^. As the French cancer registries do not include these cancers in their databases, we did not include them in the calculation of the SIR to avoid overestimation. Finally, we did not collect data in order to deal with analytic epidemiology. Some previous studies assessed for potential risk factors of developing HCL and identified some of them: farming, pesticide exposure, diesel, petrol, and ionizing radiations. Surprisingly, tobacco seems to be a protective factor in several of these studies, even if this should be confimed^57–60^.

In this 10-year follow-up analysis, we confirmed the favorable prognosis of HCL. PNAs are the best choice of treatment and result in the best CR rate, DOR, RFS, and CIR. Relapses are still an issue, with a 10-year CIR of 39%. In our cohort, there was no difference between patients who switched or did not switch between PNAs from the first to the second line. Interestingly, we found a relatively higher risk of solid cancers and hematological malignancies in HCL patients: it seems that PNAs are not a risk factor for second cancers. Few prospective studies exist for HCL patients^41^. Therefore, we plan to perform a prospective national cohort study that will include patients with HCLc, HCLv, and splenic diffuse red pulp small B-cell lymphoma (SDRPL).

## Supplementary information

Supplementary information
