# Supplementary material for: Analysis of a cohort of 279 patients with hairy-cell leukemia (HCL): 10 years of follow-up
Source: Blood Cancer J. 2020 May 27;10(5):62. doi: 10.1038/s41408-020-0328-z (PMC7253459; doi:10.1038/s41408-020-0328-z)
Supplement: Supplementary file 1 — Supplementary information [file 41408_2020_328_MOESM1_ESM.docx]

SUPPLEMENTARY INFORMATION

**Supplementary information 1. Personal history of cancer.** *ENT: Ear, Nose and Throat, DLBCL: Diffuse Large B-cell Lymphoma, CLL: Chronic Lymphocytic Leukemia, MGUS: Monoclonal Gammopathy of Undetermined Significance, MALT: Mucosa-Associated/Lymphoma Tissue.*

| **Cancer** | **n=31 malignancies** |
| --- | --- |
| **Prostate** | 8 |
| **Colorectal** | 4 |
| **Melanoma** | 3 |
| **Non melanoma skin cancer** | 2 |
| **Thyroid** | 2 |
| **Breast** | 2 |
| **MGUS** | 2 |
| **Testicular** | 1 |
| **Kaposi** | 1 |
| **Pancreas** | 1 |
| **Cervix** | 1 |
| **ENT** | 1 |
| **MALT** | 1 |
| **DLBCL** | 1 |
| **CLL** | 1 |

**Supplementary information 2. Familial history of cancer.** *MPN: Myeloproliferative Neoplasm, AML: Acute Myeloid Leukemia, MM: Multiple Myeloma***.**

| **Solid cancer** | **n=69 malignancies** |  |  |  |  |
| --- | --- | --- | --- | --- | --- |
| **Bronchopulmonary** | 12 |  |  |  |  |
| **Colorectal** | 11 |  |  |  |  |
| **Breast** | 10 |  |  |  |  |
| **Kidney** | 6 |  |  |  |  |
| **ENT** | 6 |  |  |  |  |
| **Uterus** | 4 |  |  |  |  |
| **Prostate** | 3 |  |  |  |  |
| **Unknown** | 4 |  |  | **Hematological malignancy** | **n=16 malignancies** |
| **Brain** | 3 |  |  | **Leukemia** | 5 |
| **Pancreas** | 2 |  |  | **Lymphoma** | 3 |
| **Liver** | 2 |  |  | **CLL** | 2 |
| **Gastric** | 2 |  |  | **MPN** | 2 |
| **Bone** | 1 |  |  | **AML** | 1 |
| **Cutaneous** | 1 |  |  | **HCL** | 1 |
| **Biliairy duct** | 1 |  |  | **MM** | 1 |
| **Rhabdomyosarcoma** | 1 |  |  | **MGUS** | 1 |

**Supplementary information 3.** **Flow chart.** *arterial ischemia (1), hemorrhagic shock (1), road accident (1), post-operative death (1), tamponade (1), *CR: Complete Remission, PR: Partial Remission, PD: Progressive Disease.*

**n = 279 patients**

**Death = 50 patients**

**Still alive at last follow-up**

**229 patients**


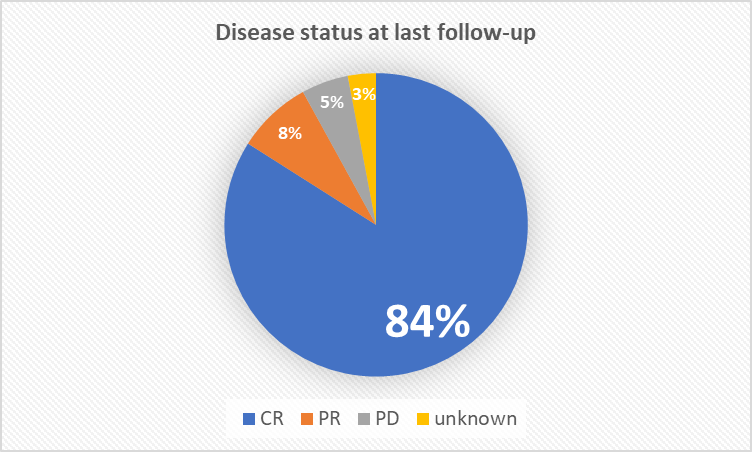


| **Cause of death** | **n = 50** |
| --- | --- |
| **Unknown** | 17 |
| **Second cancer** | 11 |
| **Progression of HCL** | 9 |
| **Infection** | 8 |
| **Other*** | 5 |

**Supplementary information 4. Response to first line treatment.** *ORR: Overall Response Rate. CR: Complete Response, other treatments including all treatments and IFNα except cladribine or pentostatin in monotherapy. *p < 0.001 (χ^2^ test) compared to cladribine and pentostatin.*

**Supplementary information 5. Median DOR according to the line of treatment.** *DOR: Duration Of Response.*

| **Line** | **Median DOR [min-max] (months)** |
| --- | --- |
| **1** | 91 [3-335] |
| **2** | 55 [0-281] |
| **3** | 45 [0-235] |
| **4** | 23 [7-343] |

**Supplementary information 6. OS, uni- and multivariate analyses.** *Cox regression analysis*. *HR: Hazard Ratio. CI-: Confidence Interval. CR1=achieving CR after first line treatment. *percentage of hairy cells at diagnosis taken as a continuous variable. **cladribine taken as reference.*

| **Overall Survival** | | | | | | | | |
| --- | --- | --- | --- | --- | --- | --- | --- | --- |
| **Univariate analysis** | | | |  | **Multivariate analysis** | | | |
| **Variables** | **p** | **HR** | **95% CI** |  | **Variables** | **p** | **HR** | **95% CI** |
| **Age at diagnosis** | < 0,001 | 1,098 | 1,065;1,133 |  | **Age at diagnosis** | < 0,001 | 1,082 | 1,044;1,121 |
| **Hairy cells at diagnosis*** | 0,635 | 0,997 | 0,983;1,011 |  | **Hairy cells at diagnosis*** | 0,733 | 1,003 | 0,988;1,018 |
| **Infection at diagnosis** | 0,099 | 1,685 | 0,906;3,133 |  | **Infection at diagnosis** | 0,270 | 1,524 | 0,720;3,225 |
| **Second cancer** | 0,192 | 1,520 | 0,810;2,850 |  | **Second cancer** | 0,782 | 1,111 | 0,525;2,352 |
| **First line treatment**** | 0,042 | 0,465 | 0,222;0,974 |  | **First line treatment**** | 0,261 | 0,600 | 0,246;1,462 |
| **CR1** | 0,452 | 0,781 | 0,409;1,489 |  | **CR1** | 0,230 | 0,602 | 0,263;1,378 |

**Supplementary information 7. RFS after first line treatment: (A) all patients, (B) according to first line treatment: cladribine, pentostatin, ‘other’ treatments including IFN, (C) according to first line treatment: cladribine, pentostatin, IFN.** *Kaplan Meier method.*


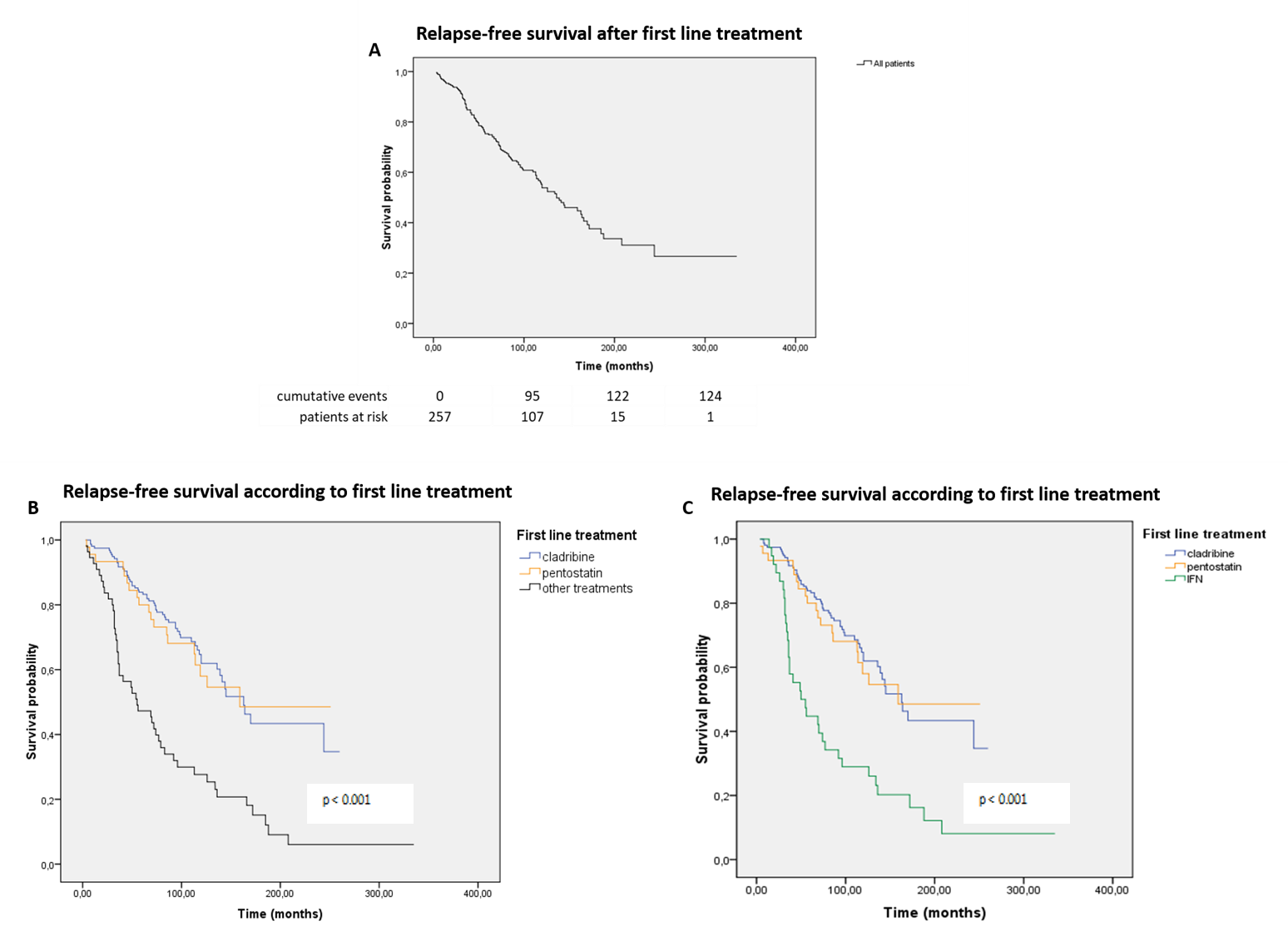


**Supplementary information 8. RFS after first line treatment, uni- and multivariate analyses.** *Cox regression analysis*. *HR=hazard ratio. CI=confidence interval. CR1=achieving CR after first line treatment. *percentage of hairy cells at diagnosis taken as a continuous variable. **’other’ treatments taken as reference.*

| **Relapse-Free Survival** | | | | | | | | |
| --- | --- | --- | --- | --- | --- | --- | --- | --- |
| **Univariate analysis** | | | |  | **Multivariate analysis** | | | |
| **Variables** | **p** | **HR** | **95% CI** |  | **Variables** | **p** | **HR** | **95% CI** |
| **Age at diagnosis** | 0,239 | 0,991 | 0,975;1,006 |  | **Age at diagnosis** | 0,331 | 0,991 | 0,974;1,009 |
| **Hairy cells at diagnosis** | 0,002 | 1,011 | 1,004;1,018 |  | **Hairy cells at diagnosis** | 0,006 | 1,010 | 1,003;1,018 |
| **Infection at diagnosis** | 0,397 | 1,203 | 0,784;1,844 |  | **Infection at diagnosis** | 0,219 | 1,359 | 0,833;2,218 |
| **Second cancer** | 0,842 | 0,957 | 0,620;1,477 |  | **Second cancer** | 0,339 | 1,270 | 0,778;2,074 |
| **First line treatment**** | < 0,001 | 3,032 | 1,777;5,174 |  | **First line treatment**** | 0,004 | 2,533 | 1,349;4,754 |
| **CR1** | < 0,001 | 0,293 | 0,199;0,430 |  | **CR1** | < 0,001 | 0,355 | 0,228;0,555 |

**Supplementary information 9. RFS according to second line treatment (RFS2).** *Kaplan Meier method***.**


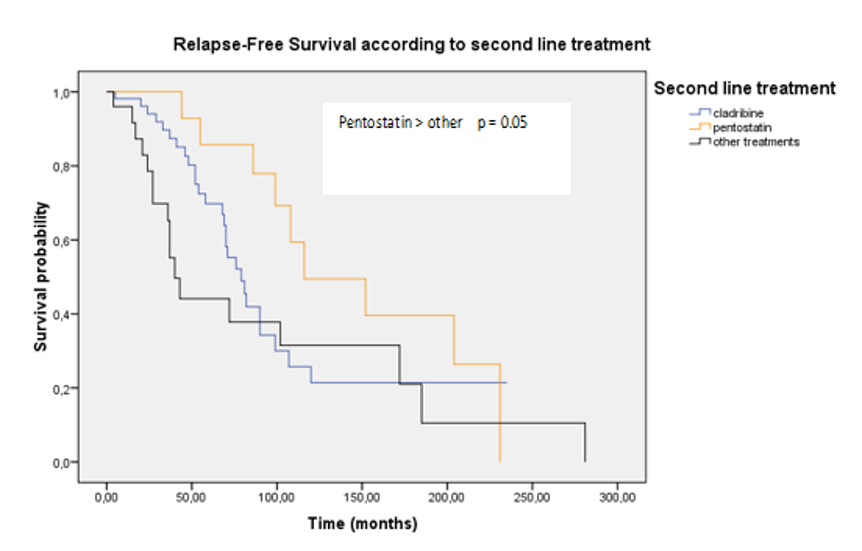


**Supplementary information 10. Patients who received PNA both in first and second lines.**

| **PNA in first and second lines** | **n=44** |
| --- | --- |
| **No switch** | **31** |
| Cladribine cladribine | 30 |
| Pentostatin pentostatin | 1 |
| **Switch** | **13** |
| Cladribine pentostatin | 8 |
| Pentostatin cladribine | 5 |

**Supplementary information 11. RFS2 for patients treated with PNA both in first and second lines**. ‘switch’ patients (black) *versus* ‘no switch’ patients (red). *Kaplan Meier method.*


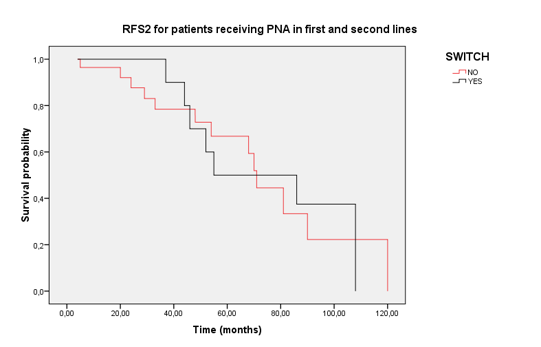


**Supplementary information 12. Cumulative incidence of second cancers, uni- and multivariate analyses: (A) all cancers, (B) solid cancers, (C) hematological malignancies.** sd*HR=subdistribution hazard ratio. CI=confidence interval. IFN=interferon alpha.*

| **A** | **All cancers** | | | | | | | | | | |
| --- | --- | --- | --- | --- | --- | --- | --- | --- | --- | --- | --- |
|  | **Univariate analysis** | | | | |  | **Multivariate analysis** | | | | |
|  | **Variables** | **p** | **sdHR** | **95% CI** | |  | **Variables** | **p** | **sdHR** | **95% CI** | |
|  | **Age at diagnosis** | 0,006 | 1,030 | 1,010;1,050 | |  | **Age at diagnosis** | 0,063 | 1,020 | 0,999;1,042 | |
|  | **Familial history of cancer** | 0,190 | 1,460 | 0,827;2,580 | |  | **Familial history of cancer** | 0,150 | 1,521 | 0,859;2,691 | |
|  | **Personal history of cancer** | 0,100 | 1,820 | 0,886;3,730 | |  | **Personal history of cancer** | 0,530 | 1,291 | 0,585;2,845 | |
|  | **Cladribine** | 0,078 | 0,632 | 0,379;1,050 | |  | **Cladribine** | 0,120 | 0,627 | 0,347;1,133 | |
|  | **Pentostatin** | 0,750 | 0,916 | 0,539;1,560 | |  | **Pentostatin** | 0,380 | 0,764 | 0,418;1,396 | |
|  | **IFN** | 0,013 | 0,468 | 0,257;0,852 | |  | **IFN** | 0,038 | 0,529 | 0,290;0,966 | |
|  |  |  |  |  |  |  |  |  |  |  |  |
|  |  |  |  |  |  |  |  |  |  |  |  |
|  |  |  |  |  |  |  |  |  |  |  |  |
| **B** | **Solid cancers** | | | | | | | | | | |
|  | **Univariate analysis** | | | | |  | **Multivariate analysis** | | | | |
|  | **Variables** | **p** | **sdHR** | **95% CI** | |  | **Variables** | **p** | **sdHR** | **95% CI** | |
|  | **Age at diagnosis** | 0,068 | 1,020 | 0,999;1,030 | |  | **Age at diagnosis** | 0,098 | 1,018 | 0,997;1,040 | |
|  | **Familial history of cancer** | 0,033 | 1,950 | 1,050;3,610 | |  | **Familial history of cancer** | 0,017 | 2,117 | 1,146;3,910 | |
|  | **Personal history of cancer** | 0,530 | 0,688 | 0,213;2,220 | |  | **Personal history of cancer** | 0,300 | 0,500 | 0,134;1,870 | |
|  | **Cladribine** | 0,850 | 0,943 | 0,518;1,710 | |  | **Cladribine** | 0,420 | 0,747 | 0,367;1,520 | |
|  | **Pentostatin** | 0,100 | 0,594 | 0,320;1,110 | |  | **Pentostatin** | 0,093 | 0,541 | 0,265;1,110 | |
|  | **IFN** | 0,090 | 0,563 | 0,290;1,090 | |  | **IFN** | 0,170 | 0,618 | 0,310;1,230 | |
|  |  |  |  |  |  |  |  |  |  |  |  |
|  |  |  |  |  |  |  |  |  |  |  |  |
|  |  |  |  |  |  |  |  |  |  |  |  |
| **C** | **Hematological malignancies** | | | | | | | | | | |
|  | **Univariate analysis** | | | | |  | **Multivariate analysis** | | | | |
|  | **Variables** | **p** | **sdHR** | **95% CI** | |  | **Variables** | **p** | **sdHR** | **95% CI** | |
|  | **Age at diagnosis** | 0,012 | 1,060 | 1,010;1,100 | |  | **Age at diagnosis** | 0,120 | 1,035 | 0,991;1,080 | |
|  | **Familial history of cancer** | 0,460 | 0,631 | 0,186;2,140 | |  | **Familial history of cancer** | 0,500 | 0,651 | 0,189;2,250 | |
|  | **Personal history of cancer** | < 0,001 | 5,210 | 2,060;13,200 | |  | **Personal history of cancer** | 0,028 | 3,473 | 1,144;10,550 | |
|  | **Cladribine** | 0,170 | 0,526 | 0,211;1,310 | |  | **Cladribine** | 0,930 | 0,947 | 0,303;2,960 | |
|  | **Pentostatin** | 0,250 | 1,720 | 0,678;4,340 | |  | **Pentostatin** | 0,420 | 1,558 | 0,528;4,600 | |
|  | **IFN** | 0,033 | 0,234 | 0,061;0,893 | |  | **IFN** | 0,095 | 0,315 | 0,081;1,220 | |

| **Study** | **Type** | **n** | **Follow-up** | **Treatments** | **ORR** | **CR** | **RFS/PFS** | **Relapses** | **OS** |
| --- | --- | --- | --- | --- | --- | --- | --- | --- | --- |
| **Bastie et al** | retrospective | 30 | 5 years | cladribine | 96% | 86% | 66% at 5 years | 17% | 83% at 5 years |
| **Ongoren et al** | retrospective | 71 | 57 months | cladribine 31 IFN 19 splenectomy 16 rituximab 1 | 97% 84% 81% | ND | 62% (entire cohort) | 16% 53% 69% | 83% (entire cohort) |
| **Jehn et al** | retrospective | 44 | 8.5 years | cladribine | 99% | 98% | 36% at 12 years | 39% | 79% at 12 years |
| **Mandanat et al** | retrospective | 61 | 72 months | cladribine | 97% | 78% | 51% at 10 years | 31% | 93% at 10 years |
| **Gaman et al** | retrospective | 39 | 52 months | IFN 29  (with maintenance 14) | 78% (for IFN) | 14% (for IFN) | median 28 months in case of maintenance IFN |  | median 8 years |
| **Lopez Rubio et al** | retrospective | 107 | ND | cladribine 80 pentostatin 27 | 100% 100% | 88% 92% | 144 months 95 months | 25% 51% | ND |
| **Hacioglu et al** | retrospective | 94 | 28 months | cladribine (majority) | 97% (for cladribine) | 81% (for cladribine) | median 36 months | 17% (for cladribine) | 96% at 5 years (for cladribine) |
| **Else et al** | retrospective | 233 | 16 years | pentostatin 188 cladribine 45 | 96% 100% | 82% 76% | median 16 years | 47% 48% at 15 years | 78% at 15 years |

**Supplementary information 13 (Part 1). Outcomes in other HCL cohorts.** *ND=no data; TFI=treatment-free interval; TTF=time to treatment failure.* **Supplementary information 13 (Part 2). Outcomes in other HCL cohorts.** *ND=no data; TFI=treatment-free interval; TTF=time to treatment failure.*

| **Study** | **Type** | **n** | **Follow-up** | **Treatments** | **ORR** | **CR** | **RFS/PFS** | **Relapses** | **OS** |
| --- | --- | --- | --- | --- | --- | --- | --- | --- | --- |
| **Zinzani et al** | retrospective | 121 | 105 months | cladribine 75 pentostatin 10 IFN 33 splenectomy 3 | 95% | 77% | ND | 44% | 88% |
| **Rosenberg et al** | retrospective | 88 | 251 months | cladribine | 100% | 88% | median 56 months | 58% | median 251 months |
| **Johnston et al** | prospective | 28 | 118 months | pentostatin | 100% | 89% | median 119 months | 32% | 82% |
| **Getta et al** | retrospective | age < 40: 63 age > 40: 268 | 69 months | cladribine | 98% 94% | 87% 83% | ND | ND | 100% 82%  at 10 years |
| **Maloisel et al** | retrospective | 238 | 64 months | pentostatin | 96% | 79% | 69% at 10 years | 15% | 89% at 10 years |
| **Goodman et al** | retrospective | 209 | >7 years | cladribine | 100% | 95% | median 42 months | 37% | 97% |
| **Flinn et al** | prospective | 241 | 9.3 years | pentostatin 154 pentostatin after IFN failure 87 | ND | 72% | 67% at 10 years | 18% | 81% at 10 years |
| **Saven et al** | retrospective | 358 | 58 months | cladribine (majority) | 98% | 91% | 19% at 48 months | 26% | 96% at 48 months |
